# The novel role of β-aescin in attenuating CCl_4_-induced hepatotoxicity in rats

**DOI:** 10.1080/13880209.2016.1275023

**Published:** 2017-01-09

**Authors:** Harsimran Singh, Shabir Sidhu, Kanwaljit Chopra, M. U. Khan

**Affiliations:** a Department of Research, Innovations & Consultancy, IKG Punjab Technical University, Kapurthala, Punjab, India;; b Sri Sai College of Pharmacy, Badhani, Pathankot, Punjab, India;; c Department of Life Sciences, Punjab Institute of technology, IKG Punjab Technical University, Kapurthala, Punjab, India;; d Pharmacology Research Laboratory, University Institute of Pharmaceutical Sciences, UGC Centre of Advanced Study, Punjab University, Chandigarh, India

**Keywords:** Carbon tetrachloride, Silymarin, antifibrotic, oxidative stress, nitrosative stress, *Aesculus hippocastanum*

## Abstract

**Context:** β-Aescin has anti-inflammatory, anti-oxidant and antiedematous properties.

**Objective:** The present study investigated the hepatoprotective effect and underlying mechanisms of β-aescin in CCl_4_-induced liver damage.

**Materials and methods:** Thirty-five Wistar rats were divided into six groups: normal control, CCl_4_ control, silymarin (50 mg/kg, *p.o*) and β-aescin (0.9, 1.8 and 3.6 mg/kg, *i.p.*) treatment for 14 d. CCl_4_ (1 mL/kg, *i.p.* for 3 d) was administered to produce hepatic damage. Ponderal changes and liver marker enzymes were estimated. Hepatic oxidative and nitrosative stress was estimated by levels of thiobarbituric acid reactive substances (TBARS), glutathione (GSH) and nitrite/nitrate. Serum TGF-β1 and TNF-α were estimated by ELISA technique. Hepatic collagen and histopathological studies were carried out.

**Results:** β-Aescin (3.6 mg/kg) markedly decreased CCl_4_-induced increased levels of ALT, AST, ALP (71.77 versus 206.7, 71.39 versus 171.82, 121.20 versus 259 IU/L, respectively), total bilirubin (0.41 versus 1.35 mg/dL), TBARS (2.0 versus 8.83 nmol MDA/mg protein), nitrite/nitrate (352.50 versus 745.15 μg/mL) and increased CCl_4_-induced decreased GSH levels (0.095 versus 0.048 μmol/mg protein). β-Aescin (3.6 mg/kg) induced focal regenerative changes in liver and markedly decreased TBARS (2.0 versus 8.83 nmol MDA/mg protein), nitrite/nitrate (352.50 versus 745.15 μg/mL), TGF-β1 (92.28 versus 152.1 pg/mL), collagen content (110.75 versus 301.74 μmol/100 mg tissue) and TNF-α (92.82 versus 170.56 pg/mL) when compared with CCl_4_ control.

**Discussion and conclusion:** The findings suggest that β-aescin has a protective effect on CCl_4_-induced liver injury, exhibited via its anti-inflammatory, antioxidative, antinitrosative and antifibrotic properties inducing repair regeneration of liver. Hence, it can be used as a promising hepatoprotective agent.

## Introduction

The liver is the largest glandular and vital organ responsible for metabolism of drugs. It plays an instrumental role in detoxifying and excreting numerous xenobiotics and therapeutic agents (Ingawale et al. 2014). Injury or destruction to its functions has severe implication to the health of affected person. Administration of carbon tetrachloride (CCl_4_) induces hepatotoxicity by generating trichloromethyl (CCl_3_^−^), a free radical from dehalogenation of CCl_4_ by cytochrome (CYP-2E1). This free radical reacts with oxygen to form trichloromethylperoxy (CCl_3_OO^−^) and stimulates oxidative stress resulting in calcium homeostasis and leading to apoptosis and necrosis (Boll et al. 2001). Further, CCl_4_-induced hepatotoxicity involves the overproduction of inducible nitric oxide synthase (iNOS) generated nitric oxide in liver and enhances nitrosative stress and leads to tissue damage (Upur et al. 2009). Moreover, activation of Kupffer cells initiates a cascade of inflammatory mediators by up regulating the expression of nuclear factor kappa B (NF-κB) and subsequently augmenting levels of tumour necrosis factor-α (TNF-α), interleukin (IL)-1β, IL-6, etc. (Ma et al. 2014). In addition, CCl_4_ administration increases the deposition of extracellular matrix proteins via increased expression of transforming growth factor-β1 (TGF-β1) and activation of stellate cells. These processes produce fibroblasts and cause hepatic fibrosis and hepatocellular carcinoma, eventually ensuing into liver failure (Huang et al. 2015).

Current available drug treatment is unable to meet the demand clinically due to lack of complete cure, numerous adverse effects, lower safety, etc. Therefore, trends are trickling towards the use of herbal drugs which has a better safety and efficacy profile. β-Aescin is the chief active constituent isolated from the horse chestnut tree *Aesculus hippocastanum* L. (Hippocastanaceae). Traditionally, leaves, bark and seeds were used in the treatment of arthritis, brain trauma, stroke, venous congestion and thrombophlebitis (Celep et al. 2012). Aescin have been reported to possess anti-inflammatory, antiedamatous, antiexudative and antioxidant properties (Xiao & Wei 2005). A large number of studies have reported that decreased levels of IL-6, IL-8, vascular endothelium growth factor and inhibition of NF-*κ*B, matrix metalloproteinase (MMP) and TNF-α may be the plausible mechanisms responsible for anti-inflammatory effect of aescin (Xin et al. 2011; Wang et al. 2013). It is worthwhile to note that sodium aescinate decreases nitrosative stress by suppressing the expression of iNOS (Ji et al. 2011). In addition, aescin has been reported to protect endotoxin-induced liver injury via its anti-inflammatory and antioxidant action (Jiang et al. 2011). The current study planned to investigate the protective effect of β-aescin against CCl_4_-induced hepatotoxicity in rats and its possible mechanisms.

## Materials and methods

### Animals

Thirty-five Wistar rats (200–250 g) of either sex, procured from central animal house, University Institute of Pharmaceutical Sciences, Punjab University, Chandigarh, were employed in the present study. They were acclimatized in the institutional animal house and then maintained on rat chow (Aashirwad Industries, Mohali, India) and tap water *ad libitum*. They were exposed to normal day and night cycles. The experimental protocol used in the current study was approved by Institutional Animal Ethics Committee (IAEC) (Approval number: IAEC/504). The investigation was performed in accordance with the guidelines of the committee for the purpose of control and supervision on experimental animals (CPCSEA), India, which are in accordance with guidelines of US national institute of health (NIH) for care and use of laboratory animals.

### Drugs and chemicals

Silymarin was purchased from Tejkamal Pharmaceutical Pvt Ltd, Mumbai, India (Batch no. SIST-010). β-Aescin (E1378, >95%, powder) was purchased from Sigma Aldrich (St. Louis, MO), invoice number A/5012, Batch no. SLBC6907V. Biochemical enzymatic kits such as AST and ALT were purchased from Transasia Bio-Medicals Ltd, Solan, Himachal Pradesh, India. ALP kit was purchased from Span Diagnostics Ltd, Surat, India. Bilirubin kit was purchased from Spinreact, Girona, Spain. TGF-β1 and TNF-α ELISA kit were purchased from R&D Systems, Bio-Techne, Minneapolis, MN. All chemicals and biochemical reagents were of analytical grade and were used as freshly prepared solutions.

### Experimental design

Rats were divided into six groups, each consisting of 5–6 animals. β-Aescin was administered at a dose of 0.9, 1.8 and 3.6 mg/kg (Jiang et al. 2011). β-Aescin was dissolved in carboxymethyl cellulose (CMC) 0.5% w/v and CCl_4_ was dissolved in olive oil (1:1 ratio). Drug treatment was started on the second day of CCl_4_ administration for 14 d (Al-Qarawi et al. 2004).

Group 1: *Normal control*: Rats received normal saline *i.p.* throughout the protocol.

Group 2: *CCl_4_ control*: Rats were administered CCl_4_ (1 mL/kg, *i.p.*) for the first, second and third day.

Group 3: *Silymarin 50 mg/kg treated*: Rats administered CCl_4_ (1 mL/kg) on the first, second and third day were treated with silymarin (standard drug) at the dose of 50 mg/kg *p.o.* for 14 d.

Group 4: *β-Aescin 0.9 mg/kg treated*: Rats administered CCl_4_ (1 mL/kg) on the first, second and third day were treated with β-aescin 0.9 mg/kg *i.p.* for 14 d.

Group 5: *β-Aescin 1.8 mg/kg treated*: Rats administered CCl_4_ (1 mL/kg) on the first, second and third day were treated with β-aescin 1.8 mg/kg *i.p.* for 14 d.

Group 6: *β-Aescin 3.6 mg/kg treated*: Rats administered CCl_4_ (1 mL/kg) on the first, second and third day were treated with β-aescin 3.6 mg/kg *i.p.* for 14 d.

### Pharmacological assessment

On the day of sacrifice, rats received their respective drugs and 2 h later were injected thiopentone (50 mg/kg *i.p.*). Then the rats were given a midline incision, heart was identified and blood was drawn by cardiac puncture into a plain vial. The blood was allowed to clot and serum separated and stored at −20 °C till further use. Liver was identified, removed and rinsed with normal saline and weighed. The liver was divided into four parts. One portion of it was stored in 10% formalin for histopathological studies. Another portion was used for preparing homogenate for carrying out estimation of hydroxyproline content. The remaining tissue was used to estimate oxidative and nitrosative stress parameters.

### Ponderal changes

*Body weight*: Change in body weight was expressed as % body weight using formula:

% Body weight=Difference of final body weight and Initial body weight/final body weight×100

*Liver weight* (*relative weight*): Change in liver weight was expressed as liver weight/100 g body weight referred as relative weight of liver using formula:

Liverweight/final body weight×100

### Assessment of serum biochemical parameters

Serum levels of alanine aminotransferase (ALT), aspartate aminotransferase (AST), alkaline phosphatase (ALP), bilirubin total and direct were assessed using commercially available kits. The units of serum ALT, AST and ALP were expressed as IU/L and amount of serum bilirubin level was determined by mg/dL.

### Assessment of tissue biochemical estimations

#### Preparation of tissue homogenate

Tissue was rinsed with 0.9% ice-cold normal saline. 10% homogenate with phosphate buffer saline was prepared. Then tissue homogenate was centrifuged at 1008*g* for 20 min at 4 °C.

#### Lipid peroxidation: thiobarbituric acid reactive substances (TBARS) estimation

To 500 μL homogenate, an equal amount of PBS buffer was added and incubated for 2 h at 37 °C. After incubation, 1 mL of 10% trichloroacetic acid was added to the mixture and centrifuged at 1008*g* for 10 min. Supernatant (1 mL) was added to 1 mL of 0.67% TBA solution and boiled for 15 min on water bath and added 1 mL of distilled water after cooling under tap water. The colour intensity was determined at 532 nm and expressed as nmol of MDA/mg protein (Wills 1966).

#### Reduced glutathione (GSH)

Tissue homogenate (1 mL) was mixed with 1 mL of 4% sulphosalicylic acid and kept the solution at 4 °C for 1 h. The solution was then centrifuged at 1200*g* for 15 min at 4 °C. To supernatant (0.1 mL), 2.7 mL of phosphate buffer (0.1 M pH 8.0) and 0.2 mL of 0.01 M Ellman's reagent (DTNB: 5,5′-dithio bis-2-nitrobenzoic acid) (0.4% in phosphate buffer 0.1 M, pH 8.0) was added. The absorbance of the solution was measured at 412 nm against blank and expressed as μM/mg protein (Ellman 1959).

#### Tissue nitrite/nitrate level

Supernatant from homogenate (500 μL) and Griess reagent (1% sulfanilamide, 0.1% naphthylethylenediamine dihydrochloride, 2.5% H_3_PO_4_) followed by incubation at room temperature for 10 min. The absorbance was measured at 540 nm. The amount of nitrite was calculated from a sodium nitrite (NaNO_2_) standard curve and was expressed as μM/mg of protein (Green et al. 1982).

#### Tissue collagen level

Tissue collagen level was assessed by estimating hydroxyproline content. The frozen tissue was homogenized in 4 mL of 6 N HCl and hydrolyzed at 110 °C for 16 h. The hydrolysate was filtered, and then 30 μL aliquot of the sample was evaporated under vacuum. The sediment was dissolved in 1.2 mL of isopropanol and incubated with 0.2 mL of 0.84% chloramine-T in acetate citrate buffer (pH 6.0) for 10 min at room temperature. Then, 1.0 mL of Ehrlich’s reagent was added and the mixture was incubated at 60 °C for 25 min. The absorbance of the sample solution was measured at 560 nm and expressed as μM/100 mg tissue (Jamall et al. 1981).

#### TGF-β1 and TNF-α estimations

TGF-β1 and TNF-α were determined by ELISA method. The quantification of TGF-β1 and TNF-α was done by as per the instructions provided by R&D Systems Quantikine Rat TGF-β1 and TNF-α immunoassay kit (R&D Systems Inc., Minneapolis, MN).

#### Histopathological examination

The liver was identified and removed from the animal body. Then, major lobe of the liver was fixed in buffered 10% formalin for at least 24 h before processing followed by embedding in paraffin. Paraffin blocks were cut into 4 μm thick sections. Paraffin blocks were taken on albumin-coated slides. Haematoxylin and eosin staining of the slides was carried out for the examination of morphological changes.

### Statistical analysis

The results are expressed as mean ± SD. The data obtained were analyzed by one-way ANOVA followed by Tukey’s multiple comparison tests. The *p* value of less than 0.05 was considered to be statistically significant.

## Results

### Effect of β-aescin on ponderal changes on liver injury induced by CCl_4_ in rats

CCl_4_ caused a marked fall in % change in body weight and increase in relative liver weight (−26.36 and 7.26 g, respectively) indicating marked liver damage compared with the normal group (13.25 and 3.15 g, respectively). Administration of silymarin (standard drug) significantly increased the reduced levels of % change in body weight and decreased the elevated levels of relative liver weight as compared with the CCl_4_ group. Furthermore, silymarin administration has normalized % change in body weight and relative liver weight as compared with normal control. Treatment with β-aescin (0.9, 1.8 and 3.6 mg/kg) significantly increased the reduced levels of % change in body weight and decreased the elevated levels of relative liver weight compared with CCl_4_ control. β-Aescin at a dose of 3.6 mg/kg has almost normalized % change in body weight (4.47 g) and relative liver weight (3.86 g). The results of 3.6 mg/kg dose of β-aescin were comparable with that of silymarin. Hence, 3.6 mg/kg dose of β-aescin was most effective when compared with 0.9 and 1.8 mg/kg (Table 1).

**Table 1.** Effect of different doses of β-aescin administration on % change in body weight (g) and relative liver weight (g) after CCl_4_ challenge. Values are expressed as mean ± SD.

Groups	% change in body weight (g)	Relative liver weight/100g of body weight (g)
Normal control	13.25 ± 4.3	3.15 ± 0.16
CCl_4_ control	−26.36 ± −13.02^a^	7.26 ± 0.86^a^
Silymarin 50 mg/kg treated	4.63 ± 9.77^b^	3.932 ± 0.49^a^^,^^b^
β-Aescin 0.9 mg/kg treated	−10.126 ± 3.51^a^^,^^b^	5.99 ± 0.52^a^^,^^b^
β-Aescin 1.8 mg/kg treated	−5.52 ± 4.36^a^^,^^b^	5.38 ± 0.32^a^^,^^b^
β-Aescin 3.6 mg/kg treated	4.47 ± 4.19^a^^,^^b^	3.86 ± 0.36^a^^,^^b^

a *p* < 0.05 as compared with normal control.

b *p* < 0.05 as compared with CCl_4_ control.

### Effect of β-aescin on liver enzymes

CCl_4_ caused a marked rise in serum levels of ALT (206.40 versus 45.97 IU/L), AST (171.82 versus 44.19 IU/L), ALP (259 versus 80.30 IU/L) and bilirubin (total and direct bilirubin) (1.35 and 1.05 mg/dL versus 0.22 and 0.17 mg/dL) demonstrating a significant damage to liver compared with the normal control group. Treatment with silymarin (50 mg/kg) significantly decreased the elevated levels of ALT, AST, ALP and bilirubin in serum compared with CCl_4_ control and has normalized the same as compared with normal control. Administration of β-aescin significantly reduced levels of ALT, AST, ALP and bilirubin as compared with the CCl_4_ group. Further, β-aescin at 3.6 mg/kg dose almost normalized the levels of liver enzymes. Moreover, the effect of silymarin (50 mg/kg) was comparable in attenuation of hepatocellular damage with highest dose of β-aescin (3.6 mg/kg) (Table 2).

**Table 2.** Effect of different doses of β-aescin administration on serum ALT, AST, ALP, total bilirubin and direct bilirubin after CCl_4_ challenge. Values are expressed as mean ± SD.

Groups	ALT (IU/L)	AST (IU/L)	ALP (IU/L)	Total bilirubin (mg/dL)	Direct bilirubin (mg/dL)
Normal control	45.97 ± 8.76	44.19 ± 7.99	80.3 ± 16.21	0.21 ± 0.04	0.17 ± 0.04
CCl_4_ control	206.7 ± 18.68^a^	171.82 ± 12.19^a^	259 ± 7.03^a^	1.35 ± 0.08^a^	1.05 ± 0.06^a^
Silymarin 50 mg/kg treated	68.95 ± 9.27^a^^,^^b^	64.68 ± 6.42^a^^,^^b^	108.2 ± 4.83^a^^,^^b^	0.42 ± 0.08^a^^,^^b^	0.28 ± 0.04^a^^,^^b^
β-Aescin 0.9 mg/kg treated	152.75 ± 13.53^a^^,^^b^	128.3 ± 8.92^a^^,^^b^	194.9 ± 9.81^a^^,^^b^	0.97 ± 0.11^a^^,^^b^	0.67 ± 0.02^a^^,^^b^
β-Aescin 1.8 mg/kg treated	91.93 ± 9.57^a^^,^^b^	83.2 ± 5.67^a^^,^^b^	157.3 ± 9.39^a^^,^^b^	0.60 ± 0.04^a^^,^^b^	0.43 ± 0.03^a^^,^^b^
β-Aescin 3.6 mg/kg treated	71.77 ± 9.91^a^^,^^b^	71.39 ± 9.93^a^^,^^b^	121.2 ± 5.94^a^^,^^b^	0.40 ± 0.05^a^^,^^b^	0.27 ± 0.04^a^^,^^b^

a *p* < 0.05 as compared with normal control.

b *p* < 0.05 as compared with CCl_4_ control.

### Effect of β-aescin on hepatic collagen level

**Figure 1.**
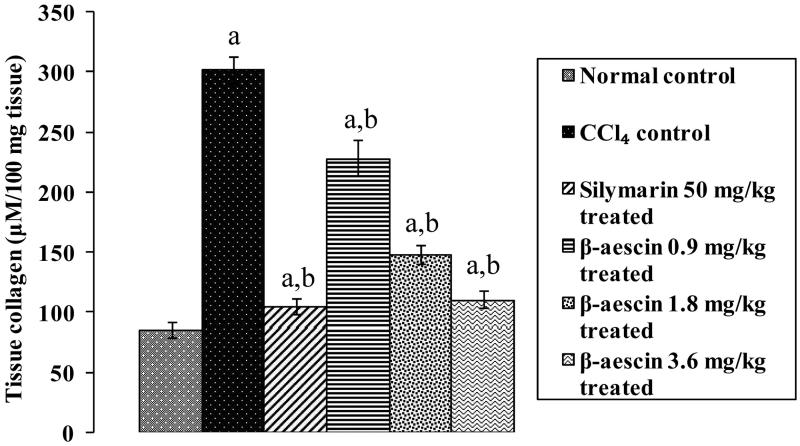
Effect of different doses of β-aescin administration on tissue collagen level after CCl_4_ challenge. Values are expressed mean ± SD. ^a^*p* < 0.05 as compared with normal control and ^b^*p* < 0.05 as compared with CCl_4_ control.

CCl_4_ caused a significant increase in tissue collagen content (301.74 versus 85.7 μmol/100 mg tissue) indicating a marked progression of hepatic fibrosis compared with the normal control group. Administration of silymarin markedly reduced the tissue collagen levels as compared with the CCl_4_ group and has normalized the levels to normal control. Treatment with β-aescin (0.9, 1.8 and 3.6 mg/kg) significantly (*p* < 0.05) and dose dependently decreased the elevated levels of collagen when compared with CCl_4_ control. β-Aescin (3.6 mg/kg) showed prominent decrease in collagen content almost normalizing the effect of CCl_4_ as compared with normal control. While the effect of highest dose of β-aescin (3.6 mg/kg) was comparable and equivalent to silymarin treated group (Figure 1).

### Effect of β-aescin on TGF-β1 level in liver

**Figure 2.**
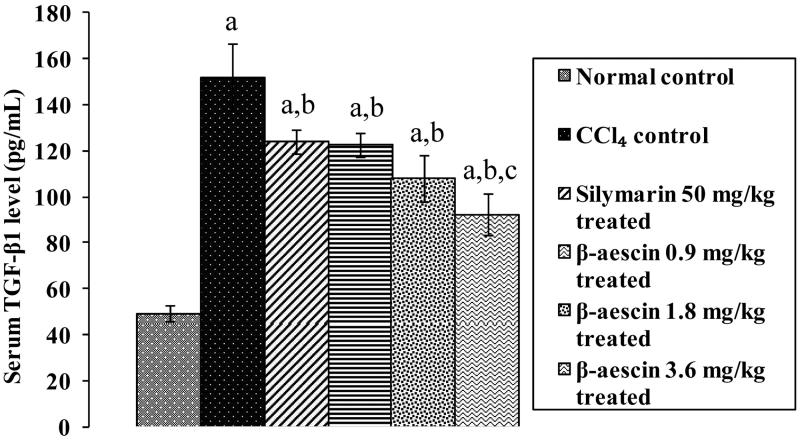
Effect of different doses of β-aescin administration on serum TGF-β1 after CCl_4_ challenge. Values are expressed mean ± SD. ^a^*p* < 0.05 as compared with normal control and ^b^*p* < 0.05 as compared with CCl_4_ control and ^c^*p* < 0.05 as compared with silymarin 50 mg/kg treated.

CCl_4_ challenge caused a marked rise serum TGF-β1 levels (152.1 versus 49.26 pg/mL) compared with normal control showing hepatic fibrosis. Administration of silymarin significantly reduced TGF-β1 levels as compared with CCl_4_. However, none of the drug treatment could recover the elevated levels of TGF-β1 induced by CCl_4_ as compared with normal control. Treatment with β-aescin significantly decreased the elevated levels of TGF-β1 when compared with CCl_4_ control. β-Aescin has showed its best effect at 3.6 mg/kg indicating antifibrotic cellular effects. It is interesting to note that β-aescin at 3.6 mg/kg has showed a significant antifibrotic effect when compared with silymarin (Figure 2).

### Effect of β-aescin on CCl_4_-induced nitrosative stress

**Figure 3.**
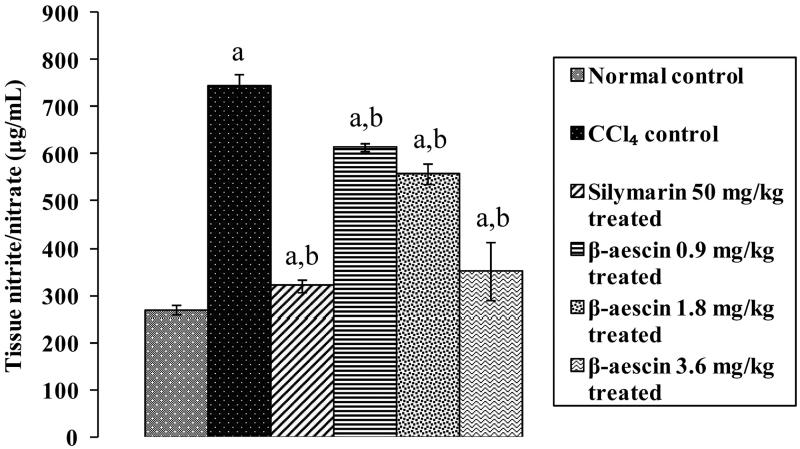
Effect of different doses of β-aescin administration on tissue nitrite/nitrate level after CCl_4_ challenge. Values are expressed mean ± SD. ^a^*p* < 0.05 as compared with normal control and ^b^*p* < 0.05 as compared with CCl_4_ control.

CCl_4_ caused a marked increase in tissue nitrite/nitrate level (745.15 versus 270.15 μg/mL) as compared with normal control. Treatment with silymarin (50 mg/kg) significantly reduced this increased level compared with the CCl_4_ group and thus has normalized the levels near to normal. β-Aescin (0.9, 1.8 and 3.6 mg/kg) administration significantly attenuated CCl_4_-induced increase in nitrite levels. Further, β-aescin (3.6 mg/kg) showed prominent decrease in close proximity to normal level against CCl_4_-induced nitrosative stress and thus is the most effective dose. The effect of β-aescin (3.6 mg/kg) was equivalent to that of silymarin (Figure 3).

### Effect of β-aescin on CCl_4_-induced lipid peroxidation

**Figure 4.**
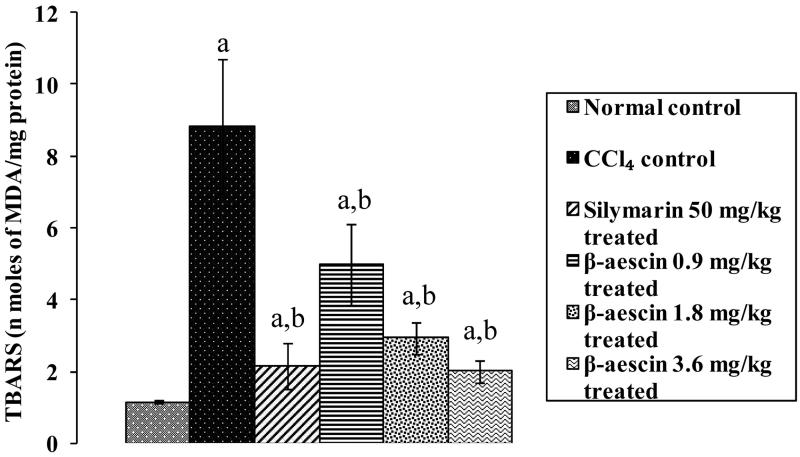
Effect of different doses of β-aescin administration on TBARS level after CCl_4_ challenge. Values are expressed mean ± SD. ^a^*p* < 0.05 as compared with normal control and ^b^*p* < 0.05 as compared with CCl_4_ control.

CCl_4_ challenge caused a marked increase in the level of lipid peroxidation (8.83 versus 1.15 nmol of MDA/mg protein) in liver as compared with normal control rats. Administration of silymarin significantly attenuated increased TBARS levels induced by CCl_4_ and has close proximity to normal control TBARS levels. Treatment with β-aescin (0.9, 1.8 and 3.6 mg/kg) significantly (*p* < 0.05) and dose dependently attenuated CCl_4_-induced increase in lipid peroxidation. The highest dose of β-aescin (3.6 mg/kg) exhibited marked decreased the level of TBARS near to the normal group indicating its antioxidant effect. The highest effective dose of β-aescin (3.6 mg/kg) has comparable results as that of silymarin (Figure 4).

### Effect of β-aescin on CCl_4_-induced changes in hepatic GSH levels

**Figure 5.**
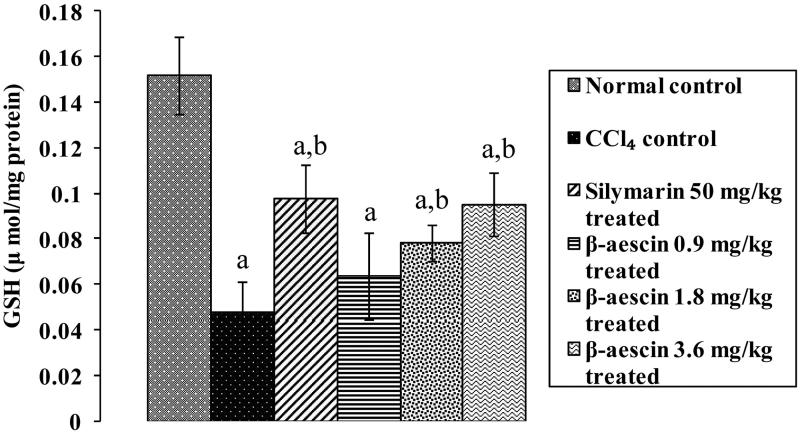
Effect of different doses of β-aescin administration on hepatic GSH level after CCl_4_ challenge. Values are expressed mean ± SD. ^a^*p* < 0.05 as compared with normal control and ^b^*p* < 0.05 as compared with CCl_4_ control.

CCl_4_ administration induced a marked reduction in hepatic GSH (0.048 versus 0.152 μmol/mg protein) as compared with normal control. Administration of silymarin (50 mg/kg) significantly increased the hepatic GSH level induced by CCl_4_ when compared with the CCl_4_ group. β-Aescin (0.9, 1.8 and 3.6 mg/kg) administration significantly increased CCl_4_-induced decrease in hepatic GSH level. Further, β-aescin (3.6 mg/kg) showed a significant increase in hepatic GSH level when compared with the CCl_4_ group and thus is the most effective dose. The effect of β-aescin (3.6 mg/kg) was equivalent to that of silymarin (Figure 5).

### Effect of β-aescin on CCl_4_-induced inflammation

**Figure 6.**
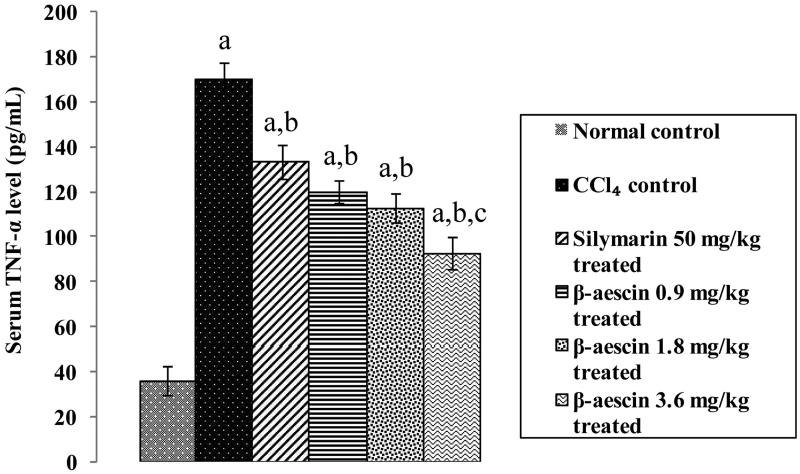
Effect of different doses of β-aescin administration on serum TNF-α after CCl_4_ challenge. Values are expressed mean ± SD. ^a^*p* < 0.05 as compared with normal control and ^b^*p* < 0.05 as compared with CCl_4_ control and ^c^*p* < 0.05 as compared with silymarin 50 mg/kg treated.

CCl_4_ caused a marked increase in serum TNF-α level (170.56 versus 36.26 pg/mL) as compared with normal control. β-Aescin (0.9, 1.8 and 3.6 mg/kg) and silymarin (50 mg/kg) significantly attenuated CCl_4_-induced increase in serum TNF-α levels. β-Aescin (3.6 mg/kg) showed marked decrease in serum TNF-α levels compared with the silymarin-treated group, indicating anti-inflammatory effect and thus demonstrating the most effective dose (Figure 6).

### Effect of β-aescin on histopathological changes on liver injury induced by CCl_4_ in rats

**Figure 7.**
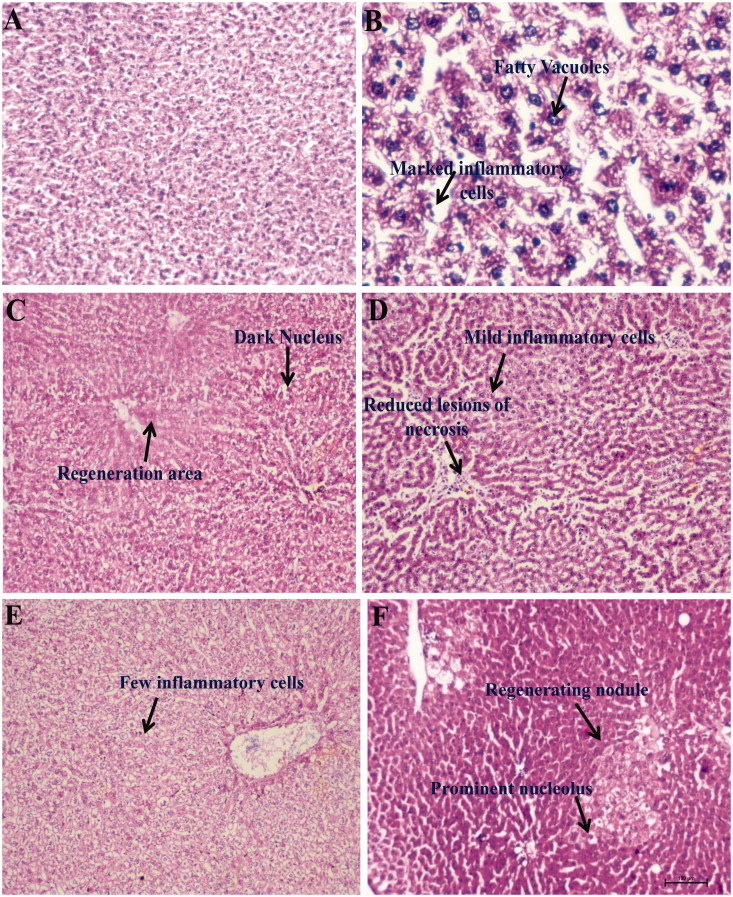
Representation of histopathological changes in livers obtained from rat groups at (H&E, ×100) (A) Normal control; (B) CCl_4_ control; (C) silymarin 50 mg/kg treated; (D) β-aescin 0.9 mg/kg treated; (E) β-aescin 1.8 mg/kg treated; (F) β-aescin 3.6 mg/kg treated.

The normal liver architecture was observed in liver histology of normal control group (Figure 7(A)). Large numbers of inflammatory cells such as lymphocytes along with hepatic sinusoidal inflammation, vacuolization of cytoplasm, fatty vacuoles, hepatocyte necrosis and devastating liver architecture have been observed in the CCl_4_ group (Figure 7(B)). Treatment with β-aescin (0.9, 1.8 and 3.6 mg/kg) and silymarin (50 mg/kg) ameliorated pathological changes induced by the CCl_4_ group in a dose-dependent manner. β-Aescin 0.9 mg/kg showed mild clusters of inflammatory cells and reduced lesions of hepatocyte necrosis (Figure 7(D)). β-Aescin 1.8 mg/kg showed very few inflammatory cells along with prominent nucleolus (Figure 7(E)). The highest dose of β-aescin (3.6 mg/kg) and silymarin (50 mg/kg) significantly attenuated the damaged liver depicting marked focal regenerative changes which are illustrated by the presence of actively dividing cells with a prominent nucleolus (Figure 7(F)). In addition, silymarin at the dose of 50 mg/kg has shown to produce hepatoprotection evidenced by the area of regeneration and dark nucleus (Figure 7(C)). The total histopathological scores of all the groups were also determined and presented in Table 3.

**Table 3.** Histopathological changes in liver of rats treated with β-aescin.

Groups	Observations	Score
Normal control	Normal architecture of liver	0
CCl_4_ control	Large numbers of inflammatory cells such as lymphocytes along with hepatic sinusoidal inflammation, vacuolization of cytoplasm, fatty vacuoles and hepatocyte necrosis	+++
Silymarin 50 mg/kg treated	Area of regeneration and dark nucleus	#
β-Aescin 0.9 mg/kg treated	Presence of less clusters of inflammatory cells and absence of hepatocyte necrosis	++
β-Aescin 1.8 mg/kg treated	Very few inflammatory lesions along with prominent nucleolus	+
β-Aescin 3.6 mg/kg treated	Marked focal regenerative changes	#

0: indicates no score is evaluated.

+++ (severe) indicates when ≥50% area of liver is affected by inflammatory cells and necrotic lesions.

++ (moderate) indicates when 25–50% area of liver is affected by inflammatory cells and necrotic lesions.

+ (mild) indicates when ≤25% area of liver is affected by inflammatory cells and necrotic lesions.

#indicates when regenerating area and prominent nucleolus has been noticed.

## Discussion

The current findings demonstrated the novel therapeutic role of β-aescin in preventing CCl_4_-induced hepatotoxicity in rats. Aescin is a natural mixture of pentacyclic triterpenoid saponin having C_55_H_86_O_24_ molecular formula (Sirtori 2001). The structure of aescin varies depending on the substitution of the hydroxyl group at C-15 and C-24 and the number and the type of the acyl group at C-16, 21, 22 and 28 (Zhang et al. 2010). Aescin exists in two forms α and β. β-Aescin is the main active constituent and is responsible for multifarious therapeutic properties (Sirtori 2001). The study design evaluated the curative effect of β-aescin after the exposure of CCl_4_. β-Aescin limited the severity of hepatotoxicity and promoted the repair and regeneration process of liver. Results of the β-aescin group were comparable with that of the silymarin-administered group further validating the beneficial effect of β-aescin.

Exposure to CCl_4_ induces hepatotoxicity by reduction in % change of body weight and increase in relative liver weight (Uzma 2011). The results of current findings are in accordance with the previous reports. On the other hand, administration of β-aescin markedly reversed the CCl_4_-induced reduction in % change in body weight and increased relative liver weight. Numerous studies have reported that intoxication with CCl_4_ produce hepatic damage illustrated by increased levels of diagnostic markers such as ALT, AST ALP and bilirubin (total and direct) (Awaad et al. 2012; Joseph et al. 2014). This contention is supported in our study showing that administration of CCl_4_ markedly elevates the level of ALT, AST, ALP and bilirubin. Further, treatment with β-aescin attenuated CCl_4_-induced increase level of ALT, AST, ALP and bilirubin in a dose-dependent manner.

Oxidative stress plays a detrimental role in the pathogenesis of various disorders including hepatotoxicity (Ganaie et al. 2015). Growing body of evidences suggest that exposure to hepatic intoxicants like CCl_4_ are involved in the generation of reactive oxygen species and free radicals (Ganaie et al. 2015). Our study revealed that administration of CCl_4_ significantly increased the levels of TBARS and decreased the levels of reduced GSH. Further, treatment with β-aescin ameliorated CCl_4_-induced hepatotoxicity by attenuating TBARS and augmenting reduced GSH level. The increased release of nitric oxide (NO) is a key factor for the development of hepatic injury (Boll et al. 2001). The hepatotoxicant, CCl_4_ stimulates endogenous nitrogen species (Boll et al. 2001) by overproduction of iNOS-generated nitric oxide in the liver. NO interacts with superoxide (O_2_^−^) anion resulting in the production of peroxynitrite (ONOO^−^) and thus causes nitrosative stress which in turn leads to hepatic damage (Upur et al. 2009). In the present study, administration of CCl_4_ significantly increased levels of tissue nitrite. However, treatment with β-aescin ameliorated CCl_4_-induced hepatic damage by attenuating nitrite/nitrate level, which may be primarily due to decline in NO level. Result of this study for the first time demonstrated antinitrosative effect of β-aescin in CCl_4_-induced hepatic injury.

Hepatic fibrosis plays a devastating role in the pathogenesis of hepatotoxicity. Tissue collagen and TGF-β levels are the key parameters for liver fibrosis (Singh et al. 2016). It has been evidenced in earlier studies by various researchers that CCl_4_ tends to raise tissue collagen and TGF-β level (Kumar et al. 2014). This contention is supported by the results obtained in our recent study that administration of CCl_4_ increased tissue collagen and serum TGF-β1 levels (Singh et al. 2016). However, treatment with β-aescin was effective in preventing hepatotoxicity in CCl_4_-induced hepatic fibrosis by significantly decreasing the levels of collagen and TGF-β1, which explain the additional antifibrotic mechanism involved in the hepatoprotection of β-aescin. CCl_4_ causes hepatic inflammation by increasing the level of TNF-α (Jiang et al. 2011). Thus, it would be possible in the present study that the rise in level of TNF-α may be additionally involved in the development of hepatotoxicity in rats. This contention is supported by the results obtained in the present study that administration of CCl_4_ increased serum level of TNF-α. However, treatment with β-aescin prevented CCl_4_-induced inflammation by reducing the circulating level of TNF-α, which may be due to inhibition of NF-κB in hepatocytes.

The hepatoprotective effect of β-aescin was additionally confirmed by histopathological studies of the liver. Treatment with β-aescin showed marked focal regenerative changes along with the reversal of more or less normal architecture of the liver, indicating hepatoprotection.

## Conclusions

It may be concluded that treatment with β-aescin shows dose-dependent hepatoprotective effect against CCl_4_-induced hepatic damage by inhibiting oxidative and nitrosative stress, histopathological changes and exhibiting antifibrotic action. These findings suggest that β-aescin is a promising agent for preventing hepatotoxicity.
